# Analysis of somatic mutations and key driving factors of cervical cancer progression

**DOI:** 10.1515/med-2023-0759

**Published:** 2023-07-28

**Authors:** Mayinuer Niyazi, Lili Han, Sulaiya Husaiyin, Ayimila Aishanjiang, Min Guo, Gulibanu Muhaimati, Hankez Rozi, Haiyan Sun, Jing Lu, Chunhua Ma, Nuermangul Rouzi, Xiaowan Liu, Kaichun Zhu

**Affiliations:** Department of Obstetrics and Gynecology, People’s Hospital of Xinjiang Uygur Autonomous Region, Urumqi 830001, China; Department of Obstetrics and Gynecology, People’s Hospital of Xinjiang Uygur Autonomous Region, No. 91, Tianchi Road, Tianshan District, Urumqi 830001, China

**Keywords:** cervical cancer, WES, *HLA* polymorphism, HPV insertion/integration, somatic mutations

## Abstract

We investigated the somatic mutations and key driving factors of cervical cancer by whole exome sequencing . We found 22,183 somatic single nucleotide variations (SNVs) in 52 paired samples. Somatic SNVs in cervical cancer were significantly higher than those in high-grade intraepithelial lesion and low-grade squamous intraepithelial lesion groups (*P* < 0.05). C → T/G accounted for 44.12% of base substitution. Copy number variation (false discovery rate < 0.05) was found in 57 chromosome regions. The three regions with significant differences between cervical cancer and non-cervical cancer groups were 1q21.1, 3q26.33, and 13q33.1, covering genes related to tumor proliferation, differentiation, and apoptosis. The frequency of human papillomavirus (HPV) insertion/integration and the number of “*tCw*” mutations in the cervical cancer group were significantly higher than those in the non-cervical cancer group (*P* < 0.05). The total number of mutations was positively correlated with the number of “*tCw*” mutations (*R* ^2^ = 0.7967). HPV insertion/integration (OR = 2.302, CI = 1.523–3.589, *P* = 0.0005), *APOBEC* enrichment (OR = 17.875, CI = 2.117–150.937, *P* = 0.001), and HLA-B*39 in *HLA-I* (OR = 6.435, CI = 0.823–48.919, *P* = 0.0042) were risk factors for cervical cancer, while HLA-DQB1*05 in *HLA-II* was a protective factor (OR = 0.426, CI = 0.197–0.910, *P* = 0.032). Conclusively, HPV insertion/integration, *APOBEC* mutagenesis, and *HLA* polymorphisms are high-risk factors for cervical cancer and may be causes of genome instability and somatic mutations. This study provides experimental data for revealing the molecular mechanism of cervical cancer.

## 1 Introduction

There are nearly 570,000 new cases and about 310,000 deaths of cervical cancer worldwide each year [1,2]. Persistent infection of high-risk human papillomavirus (HPV) is the most important risk factor for cervical cancer [3]. Although most HPVs are cleared by the body, the uncleared HPVs will persist and inactivate the tumor suppressor genes such as *TP53* and *pRb*. Meanwhile, HPVs will also integrate into the host genome, thereby exacerbating genome instability [3,4]. The accumulation of somatic mutations and genome instability caused by persistent HPV infection are all involved in the occurrence of cervical cancer. So far, among thousands of somatic mutations in human cancer types, mutational signatures of more than 40 base substitutions and 10 genome rearrangements have been identified [4,5,6,7]. The public data of next-generation sequencing and TCGA have revealed the complexity and heterogeneity of cervical lesions [8,9]. A large number of studies [10,11,12] have shown that *APOBEC3* mutagenesis is a mutational signature found in somatic mutations of a variety of cancers, especially HPV-positive cervical cancer. *APOBEC3* mutagenesis is a source of oncogenic driver events and contributes to clonal evolution and intratumor heterogeneity [12,13]. The analysis of the mutation signatures can help decipher the molecular changes and understand the precise molecular phenotype of cervical cancer, thereby contributing to the clinical diagnosis and treatment of cervical cancer [4,14].

With the increased genome data in the TCGA database and the publication of a large number of public pan-cancer studies, mutations that drive the occurrence and development of cervical cancer are constantly being identified and demonstrated. For example, the recurrent mutations of *PIK3CA*, *FBXW7*, *EP300*, *MAPK1*, *HLA-B*, *NFE2L2*, *TP53*, and *ERBB2* in cervical cancer have been confirmed [15,16]. In 2017, TCGA reported the newly identified gene mutations of *ERBB3*, *CASP8*, *HLA-A*, *SHKBP,* and *TGFBR2* in cervical cancer, and *ERBB3* (*Her3*) could be used as a therapeutic target for cervical cancer [12]. In 2019, Huang et al. reported four new significantly mutated genes, including *FAT1*, *MLL3*, *MLL2,* and *FADD*, in cervical cancer [17]. A deeper understanding of the molecular basis and the development of novel and more effective treatment modalities for cervical cancer remain unmet medical needs.

In this study, we investigated the somatic mutations and key driving factors of cervical cancer by whole exome sequencing (WES). The study subjects with cervical lesions were selected from Xinjiang, China, where there is a high incidence of cervical cancer (459/100,000–590/100,000) [18,19]. The paired cervical lesion tissue/peripheral blood samples were collected for WES. The various somatic mutations of cancer cell exomes, including single nucleotide variation (SNV) analysis, copy number variant (CNV) analysis, HPV insertion/integration analysis, APOBEC mutation mode analysis, and HLA analysis, were evaluated. Our findings may provide a deeper understanding of cervical cancer pathogenesis.

## 2 Materials and methods

### 2.1 Subjects

Patients (*n* = 52) with cervical lesions who visited the Department of Gynecology, Xinjiang Uygur Autonomous Region People’s Hospital from January 2017 to December 2019 were enrolled. All patients were positive for HPV. They were pathologically diagnosed with low-grade squamous intraepithelial lesion (LSIL), high-grade intraepithelial lesion (HSIL), or cervical cancer for the first time. None of these patients received radiotherapy or chemotherapy before sample collection. After HPV typing and pathological diagnosis, the paired fresh-frozen cervical lesion tissues and the paired peripheral blood samples were collected. This study was approved by the Ethics Committee of the People’s Hospital of Xinjiang Uygur Autonomous Region (approval number: KY2017042720), and all methods were also performed following the relevant guidelines and regulations under the committee’s supervision. Moreover, written informed consent was obtained from patients for the collection and use of samples.

### 2.2 WES

DNA extraction was performed with the magnetic bead method using the MGIEasy Magnetic Beads Genomic DNA Extraction Kit (MGI tech, Shenzhen, China). The Qubit3.0 Fluorometer (ThermoFisher, Q33216) was used for nucleic acid quantification. MGIEasy DNA Library Preparation Kit (MGIEasy, V2.0) was used to construct the library. Agilent 2100 Bioanalyzer (Agilent Technologies, G2939AA) was used to detect the size of DNA fragments, and Qubit3.0 was used to quantify the library. The Agilent SureSelect Human All Exon V7 kit (Agilent Technologies) was used to capture the whole exome region, and then the WES was performed on the MGI 2000 platform (paired ends, 150 bp), with an average sequencing depth of 64×.

### 2.3 WES data analysis

Burrows-Wheeler Aligner software (BWA, v0.7.17, http://bio-bwa.sourceforge.net/bwa.shtml) was used to map WES reads to the human reference genome hg19 (GRCh37, ftp://hgdownload.cse.ucsc.edu/goldenPath/hg19) for alignment, all of which were combined into a single tandem reference sequence. Using Fastp software (v0.20.0, http://opengene.org/fastp/fastp), the readings with quality Q20 < 90% were deleted; according to the lowest Phred quality score (MapQ), the readings with low mapping quality (MapQ < 5) were deleted. Based on the dbSNP138 database, Mill and 1000G gold standard indels database, and 1000G high confidence SNP database (all the above three databases were downloaded from ftp://ftp.broadinstitute.org/bundle/hg19) and using BaseRecalibrator module in the Genome Analysis Toolkit (GATK, v4.1.8) (https://github.com/broadinstitute/gatk/releases), the SNP correction model was constructed. The base quality in the original sequence was corrected; the systematic error caused by the sequencing instrument was eliminated; and the false positive rate of the mutation site was reduced. Based on the corrected bam file, the Mutect2 module of GATK was first used to analyze the SNP sites of the blood samples. The Genomics DBImport module was used to construct the panel of normals (PON) model of the blood samples, and the Create SomaticPanelOfNormals module was used to filter common germline mutation sites. Then, somatic analysis was performed on paired blood and cervical lesion samples using the Mutect2 module in GATK, the PoN model, and the gnomad database (http://gnomad.broadinstitute.org/downloads). After filtering out the germline mutation of each sample, the raw somatic mutation sites of the tumor samples were obtained. Finally, the FilterMutectCalls module in GATK was used to filter the mutation sites and remove the mutation sites caused by contamination, germline, and artifacts.

### 2.4 Annotation of SNV and driver gene analysis

ANNOVAR software (v2.1.1, https://annovar.openbioinformatics.org/en/latest/user-guide/download/) was used to annotate the SNVs. The amino acid and protein changes were annotated with the hg19 genome, 1,000 genomes, COSMIC mutation database (cosmic70), Clinvar database (clinvar_20200316), dbSNP150 database, and ExAC exon mutation database.

The driver genes were analyzed using MutSigCV (v1.41, https://software.broadinstitute.org/cancer/cga/sites/default/files/data/tools/mutsig/) based on the oncodriveCLUST algorithm and the oncodrive function (based on the oncodriveFML algorithm) in the R (v4.0.2, https://cran.r-project.org/bin/windows/base/old/4.0.2/) package map tools. The genes with a false discovery rate (FDR) < 0.1 was defined as the mutation driver genes.

### 2.5 Analysis of CNV

CNVkit software (V0.9.7, https://github.com/etal/cnvkit) was used to analyze the increase or decrease in copy number for a large fragment sequence on the exome. CNV on the tumor exome was conducted on paired blood and cervical lesion samples, using paired blood samples as the control group. Then, the segment module of CNVkit software was used to analyze the absolute copy number of the CNV region. The CNS files of the CNV test results of each group of samples were combined, and then, the GISTIC (V2.0.23, ftp://ftp.broadinstitute.org/pub/GISTIC2.0) software was used to calculate the significantly amplified or missing genomic regions in the cervical cancer group and the non-cervical cancer group (including LSIL and HSIL). The gisticChromPlot module in Maftools was used to integrate the GISTIC results and to plot a distribution map of significant CNVs (FDR < 0.05) in chromosomes. The difference in CNV with FDR < 0.05 between the two sets of samples was analyzed using SPSS, and the distribution of significant CNVs between the two sets of samples was plotted with the gisticOncoPlot module. Then, the richGO packages in the R language were used for GO enrichment analysis on the genes in the significant regions, and the cnetplot function in the enrichplot package was performed to construct a gene distribution map of the significantly enriched pathway.

### 2.6 HPV insertion/integration analysis

The sequences after fastp filtering and PCR repetition deletion were aligned with the human genome and HPV genome. Genbank (https://www.ncbi.nlm.nih.gov/genbank/) accession number was as follows: HPV16 [NC_001526.4], HPV18 [NC_001357.1], HPV31 [KX638481.1], HPV33 [HQ537707.1], HPV35 [M74117.1], HPV39 [LR862071.1], HPV51 [KT725857.1], HPV52 [HQ537751.1], HPV53 [EF546482.1], and HPV58 [HQ537777.1]. If a chimeric read sequence is aligned with both the HPV genome and the human host genome or one forward/reverse sequencing sequence of the same sequenced fragment is aligned to the human host genome and the other is aligned to the HPV genome, it is considered the HPV integration sequence. When it is a chimeric sequence, it should be aligned to at least 30 nt on the human host or HPV genome; otherwise, the reads were excluded. Subsequently, the BLASTn software (v2.7.1, ftp://ftp.ncbi.nih.gov/blast/executables/LATEST/) was used to further determine the integration sites. The Circos software (v0.69, http://www.circos.ca/software/download/circos/) was used to visualize HPV integration sites in the human genome.

### 2.7 *APOBEC* mutagenesis analysis

The mutation mode of *APOBEC* mutagenesis was defined as C → T or C → G mutation in the *tCw* motif. The plotApobecDiff function in the R package maftools was used to analyze the number of cytosine mutations in +/−20 nucleotides around the SNV site and to calculate the ratio of cytosine mutations in *tCw* mode to other cytosine mutations. The mutation site matrix and the *APOBEC* mutation enrichment score were evaluated with the trinucleotideMatrix function in the R package maftools (v2.2.0). The enrichment of *APOBEC*-induced mutations in the sample was calculated with the following formula: *APOBEC*_Enriched = [ntCw*backgroundC]/[nC*backgroundTCW]. According to the score of *APOBEC*_Enriched > 2, the samples were divided into *APOBEC*-enriched groups and non-*APOBEC*-enriched groups. The difference in the mutant genes between the *APOBEC*-enriched group and the non-*APOBEC*-enriched group was evaluated with the Fisher test.

### 2.8 *HLA* genotyping

The reads of *HLA-A*, *HLA-B*, *HLA-C*, *HLA-DPA1*, *HLA-DPB1*, *HLA-DQA1*, *HLA-DQB1*, and *HLA-DRB1* that were aligned to the human genome hg19 and those were not aligned to the hg19 genome were extracted with Samtools (v1.10, http://www.htslib.org/download/). *HLA* genotyping was performed using *HLA*-VBSeq software (http://nagasakilab.csml.org/hla/), and the two genotypes with the highest average coverage on each *HLA* locus were taken as the *HLA* genotypes of the sample. The *HLA* allele database of IMGT/*HLA* database 3.40.0 (ftp://ftp.ebi.ac.uk/pub/databases/ipd/imgt/hla/) was used.

### 2.9 Statistical analysis

All statistical analysis was performed using SPSS 18.0 software (SPSS Inc., Chicago, Illinois, USA). The chi-square test was used for comparing rates between groups. One-way analysis of variance was used for comparing means between samples. Pearson correlation analysis was used for determining correlations between continuous variables. *P* value < 0.05 was considered statistically significant. The 2 × 2 contingency tables were then used to test for the association of variants and clinical features by odds ratios (ORs) and 95% CIs. The forest plots were plotted using GraphPad Prism 8.0 (GraphPad, San Diego, California, USA).

## 3 Results

### 3.1 Basic information of patients

A total of 52 patients were included in this study. Their clinical data were shown in Table 1. They were aged 18–75 years old. There were 5 cases of LSIL, 18 cases of HSIL, and 29 cases of cervical cancer (22 cases of squamous cell carcinoma and 7 cases of adenocarcinoma). All these tumors were primary tumors.

**Table 1:** Basic information of included patients

Sample number	Age (years)	Ethnicity	Nationality	HPV type	Pathological type	Pathological type
H0360	49	Han	China	16	HSIL	—
H0528	46	Han	China	16	LSIL	—
H0517	52	Han	China	16	HSIL	—
H0439	52	Han	China	16	Cervical cancer	Squamous cell carcinoma
H0281	46	Han	China	16	Cervical cancer	Squamous cell carcinoma
H0322	46	Han	China	16	Cervical cancer	Adenocarcinoma
H0209	62	Han	China	52	Cervical cancer	Squamous cell carcinoma
H0237	65	Han	China	58	Cervical cancer	Squamous cell carcinoma
H0421	49	Han	China	39	Cervical cancer	Squamous cell carcinoma
H0074	49	Han	China	16	LSIL	—
H0076	32	Han	China	16	HSIL	—
H0224	33	Han	China	16	HSIL	—
H0507	50	Han	China	16	Cervical cancer	Adenocarcinoma
H0284	60	Han	China	18	Cervical cancer	Adenocarcinoma
H0240	63	Han	China	18	Cervical cancer	Squamous cell carcinoma
H0042	43	Han	China	18	HSIL	—
H0536	47	Han	China	16	HSIL	—
H0206	37	Han	China	16	HSIL	—
H0584	32	Han	China	16, 51	HSIL	—
H0075	33	Uighur	China	16	Cervical cancer	Squamous cell carcinoma
H0044	53	Uighur	China	58	Cervical cancer	Squamous cell carcinoma
H0084	50	Uighur	China	53	HSIL	—
H0083	43	Uighur	China	16	Cervical cancer	Squamous cell carcinoma
H0402	36	Uighur	China	31	HSIL	—
H0324	25	Uighur	China	18	Cervical cancer	Squamous cell carcinoma
H0433	60	Uighur	China	16	Cervical cancer	Adenocarcinoma
H0200	75	Uighur	China	16	Cervical cancer	Squamous cell carcinoma
H0289	53	Uighur	China	18	Cervical cancer	Squamous cell carcinoma
H0589	45	Uighur	China	16	HSIL	—
H0604	45	Uighur	China	18	Cervical cancer	Squamous cell carcinoma
H0030	58	Uighur	China	16	Cervical cancer	Squamous cell carcinoma
H0455	48	Uighur	China	35	HSIL	—
H0275	55	Uighur	China	18	HSIL	—
H0521	55	Uighur	China	16	Cervical cancer	Squamous cell carcinoma
H0041	47	Uighur	China	18	HSIL	—
H0212	54	Uighur	China	16	HSIL	—
H0203	68	Uighur	China	16	Cervical cancer	Adenocarcinoma
H0208	41	Uighur	China	16	Cervical cancer	Squamous cell carcinoma
H0595	40	Uighur	China	18	HSIL	—
H0047	63	Uighur	China	31/51/52	Cervical cancer	Squamous cell carcinoma
H0287	56	Uighur	China	18	Cervical cancer	Squamous cell carcinoma
H0489	44	Uighur	China	16	LSIL	—
H0235	55	Uighur	China	16	HSIL	—
H0067	41	Uighur	China	16	HSIL	—
H0390	33	Uighur	China	16	LSIL	—
H0416	60	Uighur	China	16	Cervical cancer	Squamous cell carcinoma
H0201	63	Uighur	China	16	Cervical cancer	Squamous cell carcinoma
H0034	31	Uighur	China	16	Cervical cancer	Adenocarcinoma
H0320	50	Uighur	China	18	Cervical cancer	Adenocarcinoma
H0288	63	Uighur	China	16	Cervical cancer	Squamous cell carcinoma
H0527	54	Uighur	China	18	LSIL	—
H0593	36	Uighur	China	33	Cervical cancer	Squamous cell carcinoma

Note: HPV, human papillomavirus; LSIL, low-grade squamous intraepithelial lesion; HSIL, high-grade intraepithelial lesion.

### 3.2 Distribution of somatic SNV in cervical lesions and driver gene analysis

**Figure 1:**
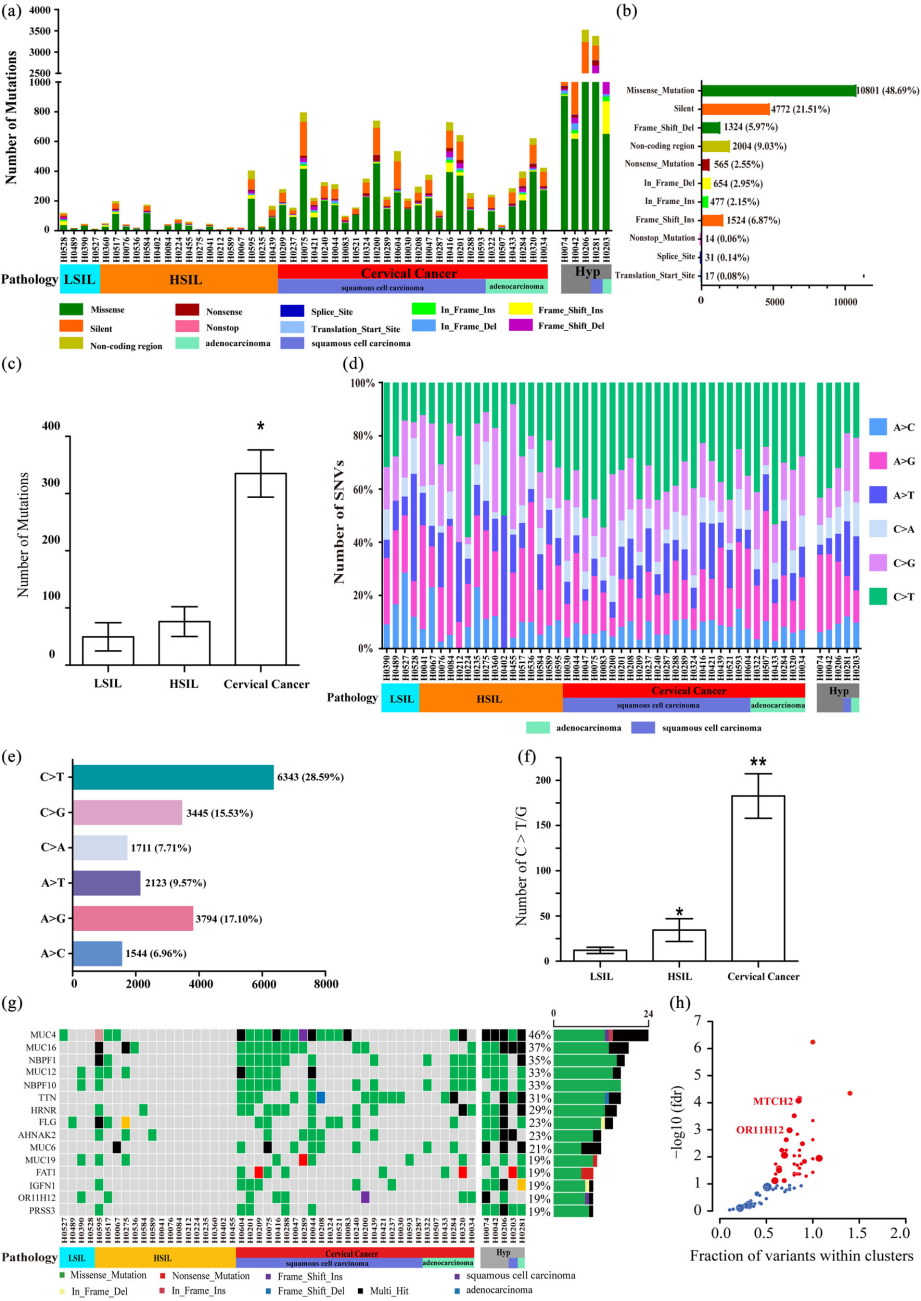
Analysis of somatic SNV. (a) The number of mutations and mutation types in 52 cervical lesion samples; (b) the total number of various non-synonymous mutation types in 52 cervical lesion samples; (c) comparison of the number of mutations in different groups of samples; (d) Percentage of SNV base changes of each sample; (e) the distribution of base mutations in 52 cervical lesion samples; (f) the number of C → T/G mutations in different groups. Compared with LSIL and HSIL, *P < 0.05, ** *P* < 0.01; (g) distribution of mutation frequency of genes in descending order. (h) Driver gene analysis using the oncodrive function in the MutSigCV and maftools package found two newly mutated genes.

There were at least 35.03 Mbp exons in the 52 pairs of cervical lesion tissue/blood samples. The median coverage depth of paired cervical lesion tissue was 64.84× (range: 24.10–132.32×), and that of paired blood samples was 64.04× (range: 24.39–123.83×) (Table S1). There were 22,183 somatic mutations in 52 paired cervical lesion tissue samples, including 2,004 non-coding regions, 4,772 synonymous mutations, 17 transcription initiation site mutations, 31 splicing site mutations, 565 nonsense mutations, 14 terminator codon mutations, 10,801 missense mutations, 1,524 insert frameshift mutations, 477 codon insertions, 654 missing codons, and 1,324 deletion frameshift mutations (Figure 1(a and b)). Among them, there were five tumor samples with abnormal mutation frequency (each samples > 1,000), and they were classified as “hypermutation” samples (Figure 1(a)). The number of SNV in the cervical cancer group (355.04 ± 42.32) was significantly higher than that in the LSIL group (49.50 ± 24.55) and the HSIL group (76.06 ± 26.00) (*P* < 0.05) (Figure 1(c)). In all tumor samples, the total mutation density was 12.18 mutations per megabase, and the total mutation density after excluding “hypermutation” samples was 6.35 mutations per megabase (Figure 1(d)). For the distribution of mutations of different bases, the total number of C → T/G mutations accounted for 44.12% of the total mutations (9788/22183) (Figure 1(e)), and the number of C → T/G mutations in the cervical cancer group (182.63 ± 24.66) was significantly higher than the LSIL group (12.00 ± 3.54) and HSIL group (34.38 ± 12.53; *P* < 0.01) (Figure 1(f)). In the 52 cervical lesion tissue samples, a total of 8,035 genes had mutations, of which 5,325 genes had missense mutations. The top 15 genes included *MUC4* (40/52, 46%), *MUC16* (19/52, 36%), *NBPF1* (18/52, 34%), *MUC12* (17/52, 32%), *NBPF10* (17/52, 32%), *TTN* (17/52, 32%), *HRNR* (16/52, 30%), *FLG* (15/52, 28%), *ANHAK2* (12/52, 23%), *MUC6* (12/52, 23%), *MUC19* (11/52, 21%), *FAT1* (10/52, 19%), *IGFN1* (10/52, 19%), *OR11H12* (10/52, 19%), and *PRSS3* (10/52, 19%) (Figure 1(g)). The frequency of mutations of these 15 genes in the cervical cancer group was significantly higher than that in the HSIL and LSIL groups (*P* < 0.05) (Figure 1(g)). Driver gene analysis using the oncodrive function in the MutSigCV and maftools package found two newly mutated genes, i.e., *OR11H12* (*q* = 0.00076) and *MTCH2* (*q* = 0.043) (Figure 1(h)).

### 3.3 Distribution of somatic CNV in cervical lesions

**Figure 2:**
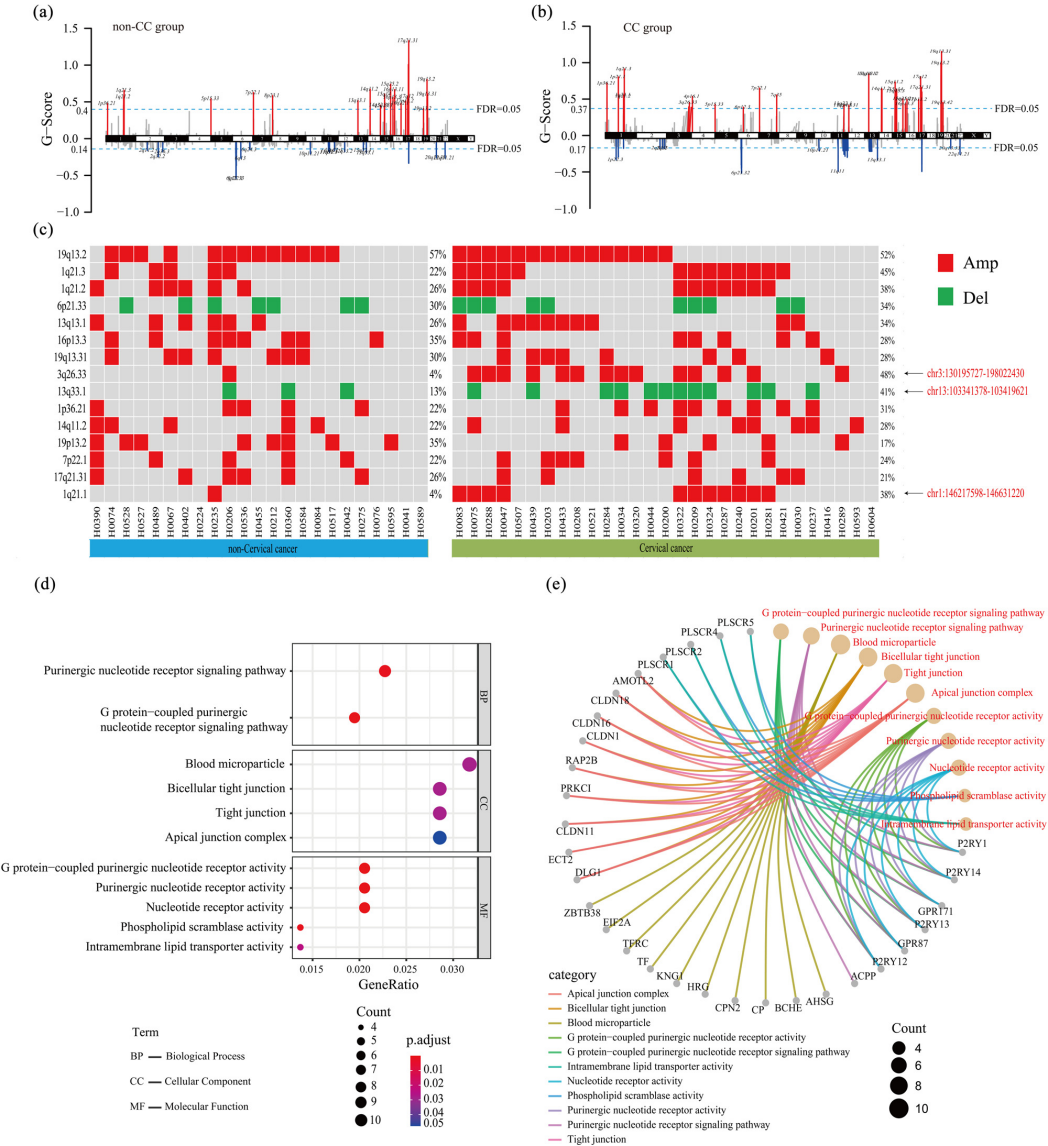
Analysis of somatic CNV (a and b): The distribution of CNVs on chromosomes in (a) non-cervical cancer and (b) cervical cancer groups Red represents amplification, blue represents deletion, and the light blue dashed line represents FDR = 0.05. (c) The top 15 chromosome regions with high CNV frequency are shown. The red square represents amplification, and the green square represents deletion. The arrows on the right indicate the three regions with significant differences between the two groups. (d) The gene enrichment at the molecular function, biological process, and cellular component levels (FDR < 0.05, *P* < 0.05) using enrichGO enrichment analysis. The size of the dots in the figure represents the number of genes in the signal pathway, and the corresponding horizontal axis is the percentage of the number of genes in the total number of genes in the signal pathway. The color represents the significance of the enriched term. (e) Significantly enriched pathways and the genes contained in each pathway. Red font indicates the name of the pathway, different line colors represent different pathways, and the size of the dots represents the number of genes in the pathway.

A total of 57 chromosome regions had CNVs (FDR < 0.05), of which there were 35 amplified-type and 22 deleted-type CNVs (Figure 2(a and b)). The chromosome region with the highest incidence of CNVs in both cervical cancer and non-cervical cancer groups was 19q13.2. Among the top 15 chromosome regions with high CNVs frequency, there were three regions with significant differences between the two groups, including 1q21.1 (*P* = 0.007, OR = 8.724 (1.213–62.722)), 3q26.33 (*P* = 0.001, OR = 11.103(1.574–78.320)), and 13q33.1 (*P* = 5.018, OR = 3.172(1.014–9.926)) (Figure 2(c)). The amplified region 1q21.1 was the chr1:146217598–146631220 region. The CNV region was 414 kb in length, and only two genes *PRKAB2* and *LOC728989* were detected. The amplified region 3q26.33 was chr3:130195727–198022430, and the length of the CNV region was 67 Mb. A total of 425 genes were detected. The deletion region 13q33.1 was chr13:103341378–103419621, with the CNV region of 78 kb in length, and only the *CCDC168* gene was detected. Then, we selected three genes with significant differences in the CNV region and analyzed their enrichment at the molecular function, biological process, and cellular component levels (FDR < 0.05, *P* < 0.05) using enrichGO enrichment analysis (Figure 2(d and e)). The results showed that these genes were closely related to tumor proliferation, differentiation, and apoptosis (such as the *P2RY* family, *PLSCR* family, *CLDN* family, etc.).

### 3.4 HPV insertion/integration at the somatic level in cervical lesions

**Figure 3:**
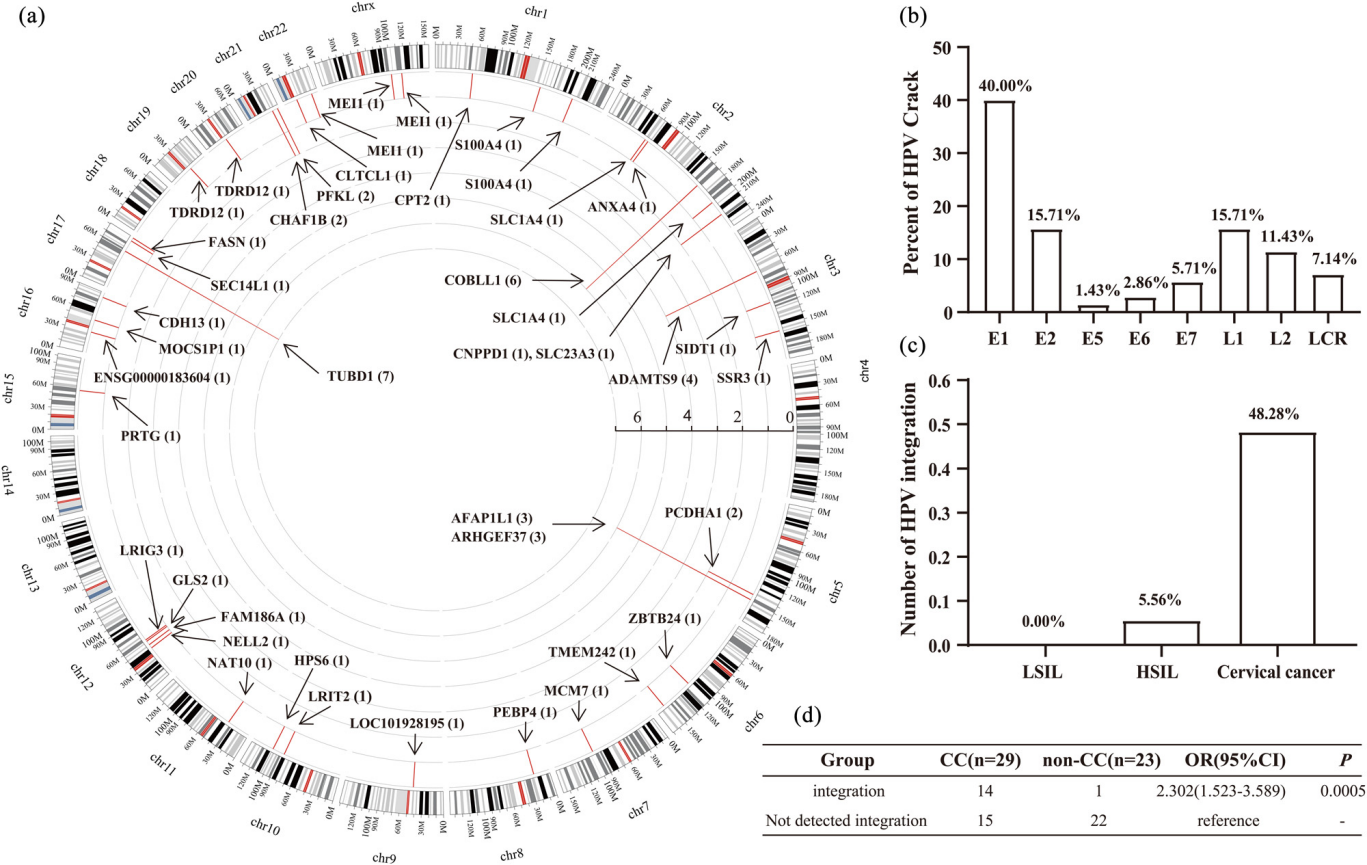
Analysis of HPV insertion/integration in cervical lesions. (a) HPV integration into the host genome. The arrows indicate the HPV integration positions on all exons. (b) The distribution of HPV breakage sites in different regions of the HPV genome. (c) HPV insertion/integration frequency analysis in 52 cervical lesion samples. (d) Relative risk analysis of HPV insertion/integration in cervical cancer.

A total of 70 HPV insertion/integration sites were identified in 52 cervical lesion samples, which were discretely distributed on the human exome (Figure 3(a)). The number of HPV insertion/integration sites in the cervical cancer group was 64/70 (91.43%) and in the HSIL group was 6/70 (8.57%). There was no HPV insertion/integration in the LSIL group (0/70, 0.00%). The HPV genome breakage sites were mainly distributed in the E1/E2 region (55.71%, 39/70) and L1 region (15.71%, 11/70) of the HPV genome (Figure 3(b)). There were a few breakage sites in the E5/E6/E7/L2/LCR of the HPV genome. For the integration into the host genome, HPV insertion/integration was not found in LSIL samples (0/5), but it was found in 5.56% (1/18) of HSIL samples and 48.28% (14/29) of cervical cancer samples (Figure 3(c)). Furthermore, it was found that HPV integration was a risk factor for cervical cancer (OR = 2.302, CI = 1.523–3.589, *P* = 0.0005) (Figure 3(d)). Compared with LSIL/HSIL samples, cervical cancer samples had more insertion sites, and these insertion sites may be related to the occurrence and development of tumors. In addition, the insertion sites in cervical cancer samples may be specifically enriched in certain genes or pathways that are related to the occurrence and development of cancer. It is speculated that the genome instability caused by HPV insertion/integration may be one of the driving factors for the occurrence and development of cervical cancer.

### 3.5 *APOBEC*-induced mutations in cervical lesions

**Figure 4:**
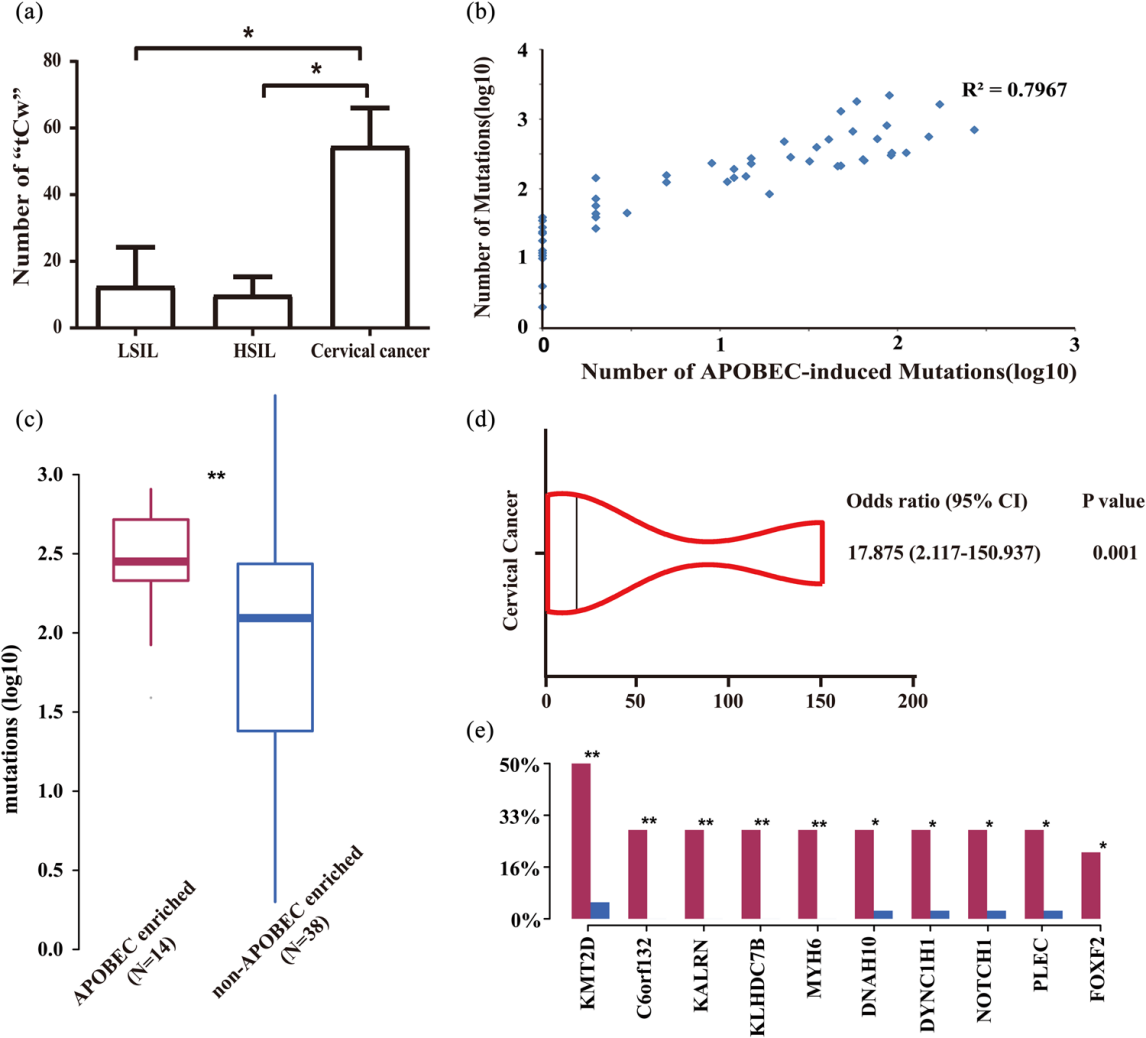
Analysis of APOBEC-induced mutations in cervical lesions. (a) Comparison of the number of “*tCw*” mutations in different groups. (b) Correlation analysis between the number of “*tCw*” mutations and the total number of mutations. (c) APOBEC enrichment analysis. (d) Forest plot of APOBEC enrichment and relative risk of cervical cancer. (e) Genes with a significant difference in mutation frequency between the APOBEC-enriched group and non-APOBEC-enriched group. Compared between the APOBEC-enriched group and the non-APOBEC-enriched group, *P < 0.05, **P < 0.01.

As shown in Figure 4(a), the number of “*tCw*” mutations in the cervical cancer group was significantly higher than that in the LSIL and HSIL groups (*P* = 0.0019). The total number of mutations was positively correlated with the number of “*tCw*” mutations (*R* ^2^ = 0.7967) (Figure 4(b)). Through the analysis with the trinucleotideMatrix function in the R package map tools (v2.2.0), 52 cervical cancer samples were divided into *APOBEC*-enriched group (14 cases) and non-*APOBEC*-enriched group (38 cases) (Figure 4(c)). Of note, 13 of the 14 *APOBEC*-enriched samples were cervical cancer samples. The remaining 1 *APOBEC*-enriched sample was HSIL. Among all SNVs in the sample, the proportion of “*tCw*” mutations in the *APOBEC*-enriched group accounted for 23.82%, which was significantly higher than that in the non-*APOBEC*-enriched group (5.77%; *P* < 0.05). Additionally, the *APOBEC* enrichment was a risk factor for the occurrence of cervical cancer (OR = 17.875, CI = 2.117–150.937, *P* = 0.001) (Figure 4(d)). The mutation frequency of ten genes in *APOBEC*-enriched group was significantly higher than that in the non-*APOBEC*-enriched group, namely *TMT2D*, *C6orf132*, *KALRN*, *KLHDC7B*, *MYH6*, *DNAH10*, *DYNC1H1*, *NOTCH1*, *PLEC,* and *FOX2* genes (*P* < 0.05) (Figure 4(e)). Among them, *C6orf132*, *KALRN*, *KLHDC7B*, *MYH6,* and *FOXF2* only had mutations in the *APOBEC*-enriched group.

### 3.6 Association analysis between HLA class I/II alleles and cervical cancer

**Figure 5:**
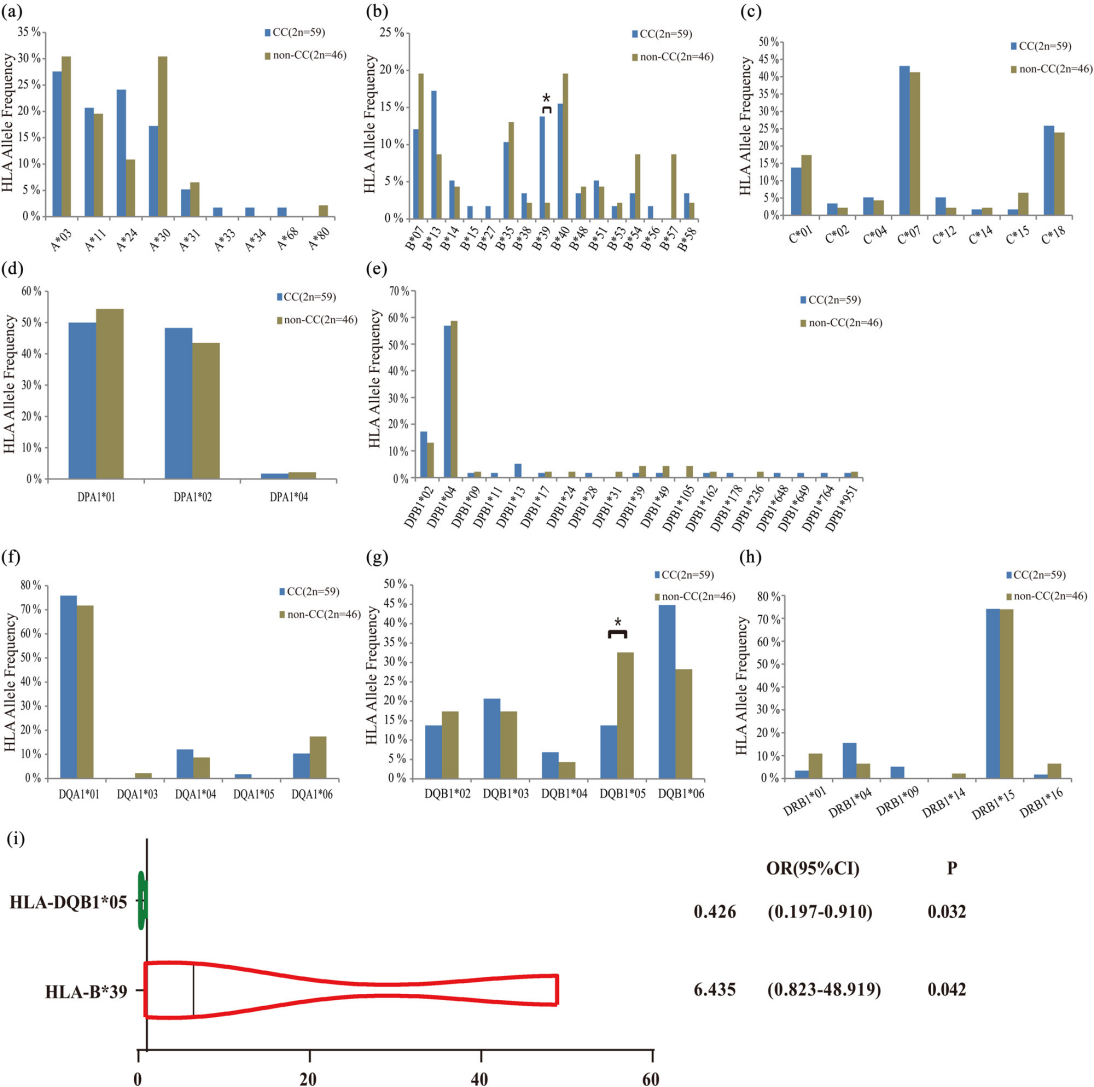
Analysis of HLA alleles. The distribution of genotypes on different *HLA* loci between cervical cancer and non-cervical cancer groups. (a) *HLA-A* genotype distribution; (b) *HLA-B* genotype distribution; (c) *HLA-C* genotype distribution; (d) *HLA-DPA1* genotype distribution; (e) *HLA-DPB1* genotype distribution; (f) *HLA-DQA1* genotype distribution; (g) *HLA-DQB1* genotype distribution; (h) *HLA-DRB1* genotype distribution; (i) forest plot of HLA-B*39 and HLA-DQB1*05 and risk of cervical cancer.

The results of HLA class I/II allele typing of 52 patients showed that in HLA class I, a total of 33 genotypes were detected in *HLA-A*, *HLA-B,* and *HLA-C* loci (Figure 5(a–c)). In *HLA* class II, a total of 38 genotypes were detected in *HLA-DPA1*, *HLA-DPB1*, *HLA-DQA1*, *HLA-DQB1,* and *HLA-DRB1* loci (Figure 5(d–h)). In *HLA-I*, HLA-B*39 was significantly different between the non-cervical cancer group and the cervical cancer group (Figure 5(i)). Moreover, HLA-B*39 was identified as a risk factor for cervical cancer (OR = 6.435, CI = 0.823–48.919, *P* = 0.0042) (Figure 5(i)). In *HLA-II*, HLA-DQB1*05 was significantly different between the non-cervical cancer group and the cervical cancer group (Figure 5(i)). Importantly, HLA-DQB1*05 was a protective factor for cervical cancer (OR = 0.426, CI = 0.197–0.910, *P* = 0.032) (Figure 5(i)).

## 4 Discussion

Persistent HPV infection is the main risk factor for the occurrence and development of cervical cancer. Whether the genome instability caused by HPV insertion/integration will lead to the occurrence and accumulation of somatic mutations in cervical cancer has attracted much attention. A comprehensive understanding of the various mutation types of somatic cells, analysis of mutation signatures, and identification of new driver genes have important guiding significance in the diagnosis and treatment of cervical cancer. Some important and common mutations including oncogenes (*PIK3CA*, *EGFR*, and *KRAS*) and suppressor genes (*PTEN*, *TP53* and *STK11,* and *MAPK*) have been reported and confirmed in cervical cancers of different populations [20,21,22,23,24]. In 2017, an analysis of the molecular characteristics of the cervical cancer genome found that *SHKBP1*, *ERBB3*, *CASP8*, *HLA-A,* and *TGFBR2* were significant driver gene mutations in cervical cancer and that *HLA-B*, *EP300,* and *FBXW7* driver gene mutations were newly identified [12]. These mutations may provide novel biomarkers for the early identification of cervical cancer [25,26].

This study performed WES on 52 pairs of cervical lesion tissue/blood paired samples and found that many membrane mucin genes (such as *MUC4, 6, 12, 16, 19*) had high-frequency mutations, among which *MUC4* was mutated in 46.7% of the samples. Similar to our results, Das et al. also found that the *MUC* family of cervical cancer patients in India had a large number of somatic mutations [27]. Among them, *MUC16* carried 11 somatic mutations and had the highest mutation frequency. Meanwhile, *MUC17* also had a high frequency of mutations [27]. Liu et al. analyzed the frequency of gene mutations in 31 cancers and found that among the 19 *MUC* family genes, nine genes were high-frequency mutation genes, of which four (*MUC4, MUC5B, MUC16,* and *MUC17*) were common high-frequency mutation genes [28]. *MUC4* is a membrane-bound mucin that can promote the progression of carcinogenesis. It has been confirmed that it may be a tumor prognostic biomarker [29]. However, in previous studies [12,24], no significant mutations in this family were reported. We consider that the discrepancy may be related to the differences in the genetic background of study participants. Additionally, this study found two significantly mutated driver genes MTCH2 and OR11H2, the role of which in cervical cancer has not been reported. MTCH2 is a mitochondrial outer membrane protein that regulates mitochondrial metabolism. It is shown that MTCH2 can inhibit tumor invasion in malignant gliomas [30]. However, whether MTCH2 is involved in regulating the occurrence and development of cervical cancer is unclear. OR11H2 is a member of the G protein-coupled receptor family, and there is no report on whether it is related to tumors. We will carry out a follow-up study to verify the roles of MTCH2 and OR11H2 in cervical cancer.

Marchuk et al. [31] performed WES sequencing on 672 tumor-containing samples and found a total of five pathogenic CNVs, namely, 1q21.1 deletion, 7q11.23 duplication, 15q11.2 deletion, 17p12 duplication, and trisomy 21. In a study on cervical cancer [32], 88 paired tumor samples and blood samples were analyzed. A total of 26 amplifications and 37 deletions were detected, including 3q26.31 (TERC, MECOM; 78%), 3q28 (TP63; 77%), and other CNVs. This study identified 57 regions of the chromosomes with CNVs. The frequency of CNVs in 1q21.1, 3q26.33, and 13q33.1 in the cervical cancer group was significantly higher than that in the non-cervical cancer group. GO analysis found that these three regions covered genes related to a variety of signaling pathways closely related to cancer occurrence and cell proliferation, differentiation, and apoptosis (such as the *P2RY* family, *PLSCR* family, *CLDN* family, etc.). The genomic instability caused by CNV in these regions may be a key factor responsible for cervical cancer occurrence. Our results were partially consistent with previous findings [12,31]. The discrepancy may be caused by differences in the genetic background of the study population.

Many studies [32,33] have shown that HPV integration usually disrupts the open reading frames of E1 and E2, and upregulates the expression of E6 and E7 oncogenes. E6 can bind and degrade the tumor suppressor protein P53 and the pro-apoptotic protein BAK, thereby increasing the resistance of host cells to apoptosis and allowing viral DNA replication [34]. Consistently, we found that most HPV insertion/integration sites were located in the E1/E2 region, and the number of HPV insertion/integration in the cervical cancer group was the largest (91.43%).

We also found that 55.59% of cervical lesions were C → T/G mutations, and C → T/G mutations were in line with the “*tCw*” mutations induced by *APOBEC*. Analysis of the “*tCw*” mutations revealed that the number of “*tCw*” mutations in cervical lesions was positively correlated with the total number of mutations. The number of “*tCw*” mutations in cervical cancer was significantly higher than that in the HSIL group and the LSIL group. *APOBEC* enrichment was a high-risk factor for cervical cancer. Similar to our results, there have been studies reporting that HPV sequence integration was accompanied by an increase in the expression of *APOBEC3A* during the malignant transformation of cervical cancer and significant *APOBEC* mutagenesis was shown in other HPV-related malignancies [35,36]. A study using TCGA has also shown that the amount of mutations induced by *APOBEC* in cervical cancer is significantly positively correlated with the total number of mutations, and *APOBEC* mutagenesis is the main source of mutations in cervical cancer [12].

Under the same HPV exposure conditions, the incidence of cervical cancer is different [37,38], which may be related to the difference in immune response caused by *HLA* gene polymorphism [39]. Bao et al. found that in the European population, the alleles HLA-DRB5*0101 and HLA-DRB3*9901 were risk factors for cervical cancer, and HLA-DRB3*301 was a protective factor for cervical cancer [40]. However, Chen et al. conducted a GWAS study on the cervical cancer susceptibility loci in European populations and found that HLA-DPB1*04:02 and rs3117027 G alleles were significantly associated with reduced risk of cervical cancer, while HLA-DPB1*03:01 was significantly associated with increased risk of cervical cancer [41,42]. Another GWAS study conducted by Shi et al. in the Chinese population showed that HLA-DPB1*03:01 and DPB1*04:01 were associated with cervical cancer susceptibility, while HLA-DPB1*05:01 and rs4282438 G alleles showed protective effects [43]. However, we obtained inconsistent results. It is reported that different genetic backgrounds may lead to differences in the incidence and gene mutation of cervical cancer [44]. Thus, we consider that this discrepancy may be due to differences in the genetic background of study participants. The samples included in this study were from Uighur and Han populations in Xinjiang, China, which have unique genetic backgrounds.

This study has some limitations. First, the sample size was small. Second, the follow-up information, especially that of LSIL and HIS patients, was incomplete. Due to the small sample size and incomplete patient data, a more detailed analysis of cervical cancer cannot be performed. Third, we did not further analyze the histological subtypes of cervical cancers. Further studies are warranted to verify the results.

In this study, through WES, we analyzed the genetic mutation signatures of cervical lesions at different stages. We found that HPV insertion/integration, *APOBEC* mutagenesis, and *HLA* polymorphism were high-risk factors for cervical cancer. In addition, MTCH2 and OR11H2 were identified for the first time as significantly mutated genes in cervical cancer. Our findings may provide an in-depth understanding of the molecular mechanisms of cervical cancer and may provide insights into the development of new diagnostic and therapeutic targets for cervical cancer.

## Supplementary Material

Supplementary material
